# Nodular scabies on a boy’s scrotum

**DOI:** 10.1590/0037-8682-0090-2023

**Published:** 2023-06-02

**Authors:** Qiuping Li, Xiujiao Xia

**Affiliations:** 1 Hangzhou Third People’s Hospital, Affiliated Hangzhou Dermatology Hospital, Zhejiang University School of Medicine, Department of Dermatology, Hangzhou, Zhejiang, China.

**FIGURE 1:**
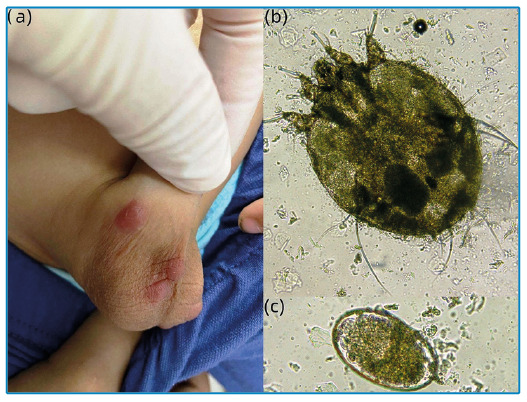
Discrete nodules on the scrotum **(a)**. Photomicroscopy of a scraping from a nodule, showing a scabies mite **(b)** and egg **(c)** (original magnification ×100).

A 3-year-old boy was brought to the hospital by his parents because of severely itchy scrotal nodules and pruritic papules on the hands that had been present for 10 days. No other family members had similar symptoms. A dermatological examination revealed papules with vesicles on the hands and discrete nodules distributed over the scrotum (Figure 1a). Microscopic examination of samples obtained by squeezing the nodules using a lancet revealed female mites (Figure 1b) and eggs (Figure 1c). After 13 days of treatment with compound sulfur cream, the papular lesions on the child’s hands were cured. However, his scrotal nodular lesions persisted, with severe itching. The lesions were then treated with topical steroids and 0.03% tacrolimus ointment. After an additional 20 days of treatment, the skin lesions completely disappeared. 

Scabies is a global public health problem that affects over 300 million people annually, with the highest prevalence among children aged under 2 years. Approximately 7-10% of patients with scabies, particularly children, develop reddish-brown infiltrated nodules that may persist for several weeks^1^. The penetration of an intact mite through the thin epidermis of flexural skin into the dermis may cause inflammatory nodules. In order to treat the condition, topical and sometimes intra-lesional corticosteroids and topical calcineurin inhibitors may be required^2^. Generally, mites and their eggs are rarely found in nodular lesions of scabies^3^. Squeeze sampling may improve the detection rate. 
